# Light-sheet fluorescence imaging to localize cardiac lineage and protein distribution

**DOI:** 10.1038/srep42209

**Published:** 2017-02-06

**Authors:** Yichen Ding, Juhyun Lee, Jianguo Ma, Kevin Sung, Tomohiro Yokota, Neha Singh, Mojdeh Dooraghi, Parinaz Abiri, Yibin Wang, Rajan P. Kulkarni, Atsushi Nakano, Thao P. Nguyen, Peng Fei, Tzung K. Hsiai

**Affiliations:** 1 Division of Cardiology, Department of Medicine, UCLA, Los Angeles, CA 90095, USA; 2 Department of Bioengineering, School of Engineering & Applied Sciences, UCLA, Los Angeles, CA 90095, USA; 3 Departments of Anesthesiology, Physiology and Medicine, Cardiovascular Research Laboratories, UCLA, Los Angeles, CA 90095, USA; 4 Division of Dermatology, Department of Medicine, UCLA, Los Angeles, CA 90095, USA; 5 California NanoSystems Institute, UCLA, Los Angeles, CA 90095, USA; 6 Department of Molecular, Cellular and Developmental Biology, UCLA, Los Angeles, CA 90095, USA; 7 Eli and Edythe Broad Center of Regenerative Medicine and Stem Cell Research, UCLA, Los Angeles, CA 90095, USA; 8 School of Optical and Electronic Information, Huazhong University of Science and Technology, Wuhan, China

## Abstract

Light-sheet fluorescence microscopy (LSFM) serves to advance developmental research and regenerative medicine. Coupled with the paralleled advances in fluorescence-friendly tissue clearing technique, our cardiac LSFM enables dual-sided illumination to rapidly uncover the architecture of murine hearts over 10 by 10 by 10 mm^3^ in volume; thereby allowing for localizing progenitor differentiation to the cardiomyocyte lineage and AAV9-mediated expression of exogenous transmembrane potassium channels with high contrast and resolution. Without the steps of stitching image columns, pivoting the light-sheet and sectioning the heart mechanically, we establish a holistic strategy for 3-dimentional reconstruction of the “digital murine heart” to assess aberrant cardiac structures as well as the spatial distribution of the cardiac lineages in neonates and ion-channels in adults.

The heart is the first mesoderm-derived functional embryonic organ after gastrulation. Embryonic stem cells play a critical role in organ and tissue development, from differentiation to proliferation to organization into the specific tissues and anatomical structures. Elucidating organ-specific differentiation of stem cells to embryonic cardiomyocytes advances the field of developmental biology^1^. By specifically labeling lineage markers and key genes with fluorescent reporters, researchers are able to visualize cardiac-specific proteins, ion-channels and signaling molecules from embryonic stem cell-derived progenitors to adult cardiomyocytes^2^. However, conventional optical microscopes are confined to image the sample with a small working distance, requiring mechanical slicing with potential risk of tearing, folding, compressing or stretching the tissue or organ, followed by 3-dimentional (3-D) reconstruction with potential under sampling^3^. Similarly, the widely used computed tomography (CT), positron emission tomography (PET), and magnetic resonance image (MRI) are limited by spatial resolution and non-specific contrast^4^,^5^.

Compared to confocal and wide-field microscopy, light-sheet fluorescence microscopy (LSFM) allows for rapid scanning with high axial resolution and low photo-bleaching, enabling spatial localization of the cellular events with multi-channels of fluorescence^6^,^7^,^8^. Initially developed to image *Caenohabditis elegans*^9^,^10^, zebrafish embryos^11^,^12^, and *Drosophilas*^13^,^14^, LSFM was confined to visualize small animal models due to the limited photon absorption and scattering. Parallel advances in optical clearing techniques^15^ have enabled us to minimize scattering of light sheet excitation and absorption by the tissues for large-scale scanning of mammalian organs such as the mouse hippocampus^16^,^17^,^18^, and mouse cochlea^19^,^20^,^21^,^22^. Our previous method followed by post-imaging synchronization algorithm has recapitulated 4-D (spatially 3-D + time) live zebrafish throughout cardiac cycles^23^,^24^,^25^, elucidating hemodynamic forces underlying cardiac trabecular formation. However, to enable rapid and replicative light-sheet imaging of intact murine hearts on a large scale, would encounter: 1) the trade-off between the light-sheet thickness and Rayleigh range for both high spatial resolution and penetration depth, 2) uneven single-sided illumination across the sample beyond the 10 mm range, 3) interference from the residual hemoglobin in the red blood cells and obstruction from the cardiac tissue, and 4) lower transmission rate of clearing solution in the intact heart in comparison with brain^26^,^27^.

In this context, and to address these considerations, we have developed a cardiac LSFM strategy for imaging the intact murine hearts on a large scale. We built a dual-sided illumination cardiac LSFM, with the aid of tuning the relative location of both illumination beams to balance the trade-off and compensate the uneven illumination for cardiac imaging across a large sample size of >10 by 10 by 10 mm^3^. We have further replaced the requirement of high numerical aperture (NA) and water-dipping objectives for high resolution with the use of a low power illumination lens^23^. This method allows for a long working distance to enable large-scale imaging with sufficient lateral and axial resolution, and avoids the need to seal the objectives in the water chamber. By adjusting the relative location of illumination beams along the propagation direction, we have imaged the whole slice without stitching image columns. Therefore, we bypassed the need to move the tissue volume or light-sheet along the propagation of the illumination to meet the beam waist^20^,^21^. Rather than using a resonant scanner to remove optical stripes and artifacts in the m-SPIM modality^28^, we have specifically applied the variational stationary noise remover^29^,^30^ as a post-processing method in our system to simplify the operation during scanning and enhance the robustness of the system. Furthermore, we have applied a simplified CLARITY method to render the heart translucent^31^, and using glycerol as a refractive index matching solution (RIMS) reduces the experimental cost. As a result, this strategy enables us to quantify the ventricular dimension, to track the cardiac lineage, and to localize the spatial distribution of cardiac-specific proteins and ion-channels from the postnatal to adult mouse hearts with sufficient contrast and resolution.

## Results

### Calibration of large-scale LSFM

**Figure 1:**
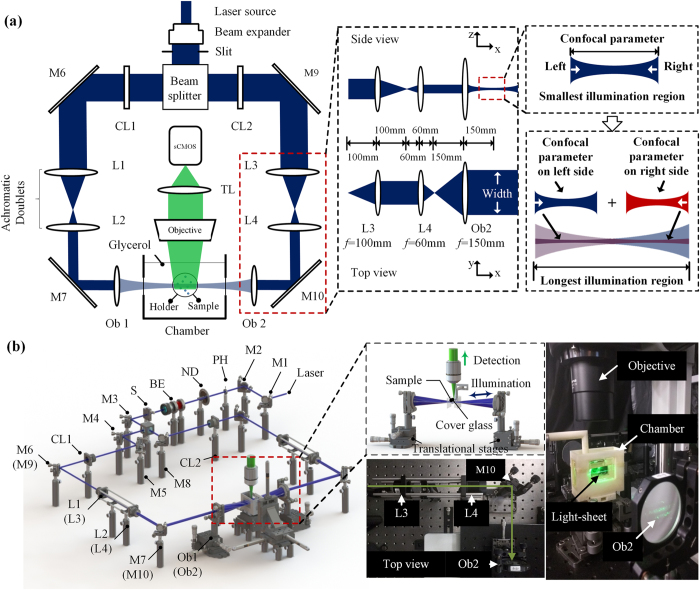
Schematic diagram of a dual-sided illumination for the light-sheet fluorescent microscope. (**a**) 2-D optical layout highlights the achromatic doublets and the chamber for mounting the samples, and illustrates a pair of beams to provide dual-sided illumination to the samples. (**b**) 3-D layout of the imaging system reveals the individual optical components. M1-10: mirror; PH: pinhole; ND: neutral density filter; BE: beam expander; S: slit; CL1-2: cylindrical lens; L1-4: achromatic doublets; Ob1-2: objective.

**Figure 2:**
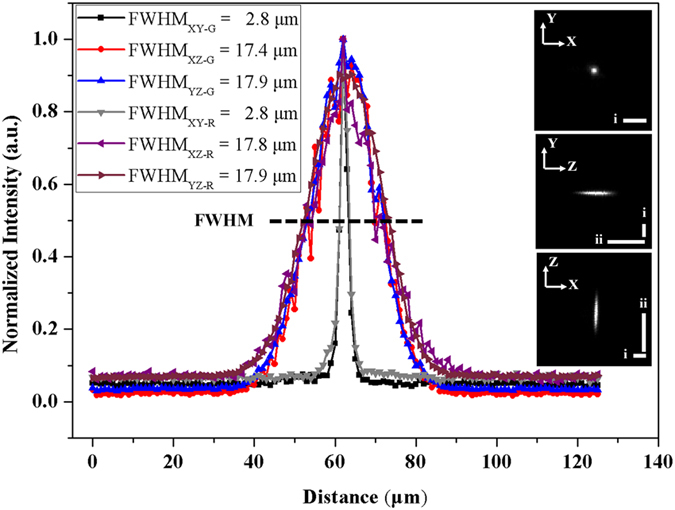
Lateral and axial resolution at the thinnest light-sheet region under the light-sheet microscope, with the 6.3X zoom-in. Values of PSF in glycerol and RIMS were shown as FWHM. R: RIMS; G: glycerol. PSFs on the *XY*-, *YZ*- and *XZ*-planes were shown with the scale bar (i–ii) 25 μm.

Figure 1 illustrates the setup of the dual-sided illumination LSFM system for localizing cardiac lineage and protein distribution. The confocal parameter was treated as the effective illumination region. Thus, for different samples, the illumination region could be tuned from the smallest (overlap of dual-sided illumination beams) to the longest region (connection of both beams) as demonstrated in Fig. 1a. Details of the system can be found in Methods and Materials. Calibration of the system was performed by measuring fluorescent beads (average size ~0.53 μm) in different configurations. We measured the point spread function (PSF) with a 6.3X zoom lens (MVX10 microscope) in both RIMS and 99.5% glycerol, and quantified the lateral and axial resolutions as the full width at half maximum (FWHM) at the beam waist. The PSF patterns of a single bead in glycerol are shown in Fig. 2. The values of lateral resolution on the *XY*-plane was ~2.8 μm in glycerol and RIMS, and the axial resolutions of *XZ*- and *YZ*-planes were ~17.4 μm and ~17.9 μm in glycerol, as well as ~17.8 μm and ~17.9 μm in RIMS, respectively. In addition, this group of comparable results demonstrated glycerol was a proper alternative for matching the refractive index, and therefore we further used glycerol as the embedding medium in the chamber.

**Figure 3:**
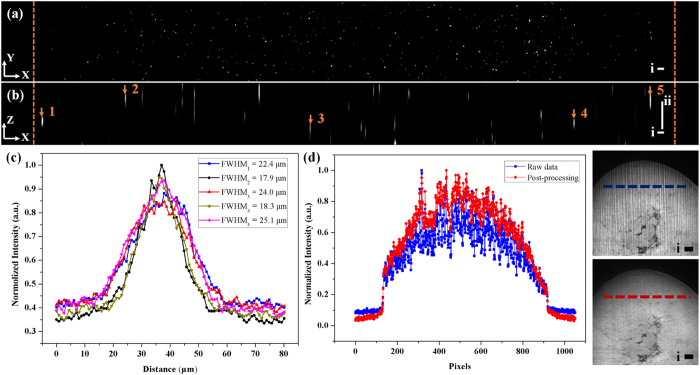
(**a,b**) Imaging raw data of beads on (**a**) *XY*- and (**b**) *XZ*-planes. Yellow dashed line is the boundary of the glass tubing. (**c**) Measured FWHM at different locations in (**b**). (**d**) Comparison between the output of the variational stationary noise remover and raw data in single pixel scale (**d**), as well as intuitive details in images. Scale bar: (i) 100 μm; (ii) 50 μm.

For quantifying the longest illumination region used for the adult heart, fluorescent beads were imaged in the same way as the sample with a 1X zoom lens for detection. In Fig. 3a,b, the yellow dashed line represents the boundary of the glass tubing in the single slice of a sectioned *XY* (a) and *XZ* (b) plane. Considering the amount of beads, the sample was assumed to be homogenous and five points were selected to test the PSF. Based on the measured values in Fig. 3c, the thinnest part of illumination was 17.9 μm while the largest one was 25.1 μm on the whole sample. All of these values are within the range of confocal parameter (25.2 μm) with the waist of 17.9 μm; therefore this strategy could be used for rapidly generating effective light-sheet illumination on the adult heart. To verify that the values in Fig. 3c were the thickness of light-sheet at different regions, we also compared the result by changing the slit size and applying wide-field illumination (Figure S1 in the Supplementary Information).

Due to photon scattering and absorption, horizontal stripes were prominent in the raw data. Based on the previous work^29^,^30^, variable stationary noise could be further removed from the genuine signal to improve signal-to-noise ratio. By specifically tuning critical parameters (noise level, lambda, angle, phase and so on) in the algorithm, two Gabor filters were applied simultaneously to eliminate stripes from the individual slices in our system. One was 20:1 (horizontal: vertical), and the other was 10:3 (horizontal: vertical). In Fig. 3d, the comparison in single pixel scale between raw data (in blue) and de-noised result (in red) was shown to indicate the fluctuation of intensity along these pixels, corresponding to the dashed lines in the cropped images of clarified mouse heart.

### Quantification of murine ventricular dimensions

**Figure 4:**
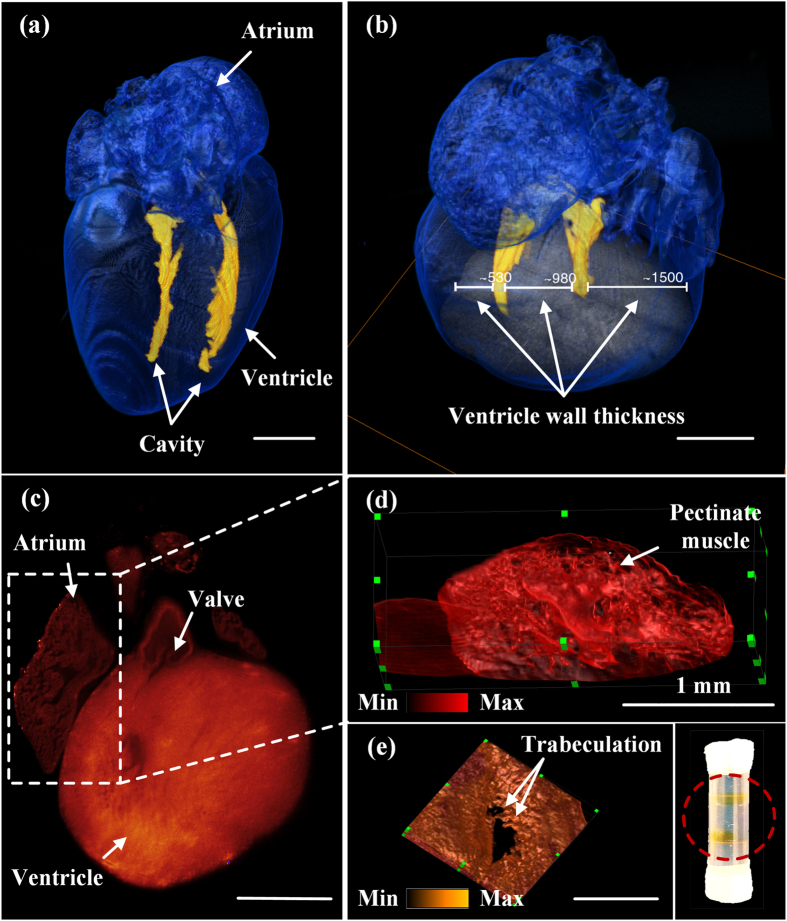
3-D architecture of a neonatal mouse heart. (**a**) 3-D rendering of the reconstructed P7 (postnatal day 7) heart (see Movie S1) reveals the small ventricular cavity in a thick wall. (**b**) The horizontal bar demarcates the left, septal, and right ventricular wall thickness at 1500 μm, 980 μm, and 530 μm, respectively. (**c**) 2-D valvular structures are visualized from a P1 mouse heart. (**d**) Pectinate muscle is prominent in the right atrium (see Movie S2). (**e**) Trabeculation is present in the ventricular endocardium (see Movie S3). The inset shows two translucent hearts after CLARITY in the tube. Scale bar: 1 mm.

The relative ventricular volume-to-wall thickness ratios provide insights into the regenerative capacity of cardiomyocyte proliferation at the neonatal stages. A wild-type postnatal heart at P7 was imaged entirely by autofluorescence at λ = 473 nm. The contour of the heart was rendered by a user-defined intensity threshold, with pixel values above this threshold being shown in blue with transparency. We manually segmented the ventricular cavity based on another intensity threshold and merged it with the blue surface. The 3-D reconstruction of the heart highlighted the ventricular cavity in yellow and the myocardium in blue (Fig. 4a, Movie S1 in the Supplementary Information). For 3-D rendering, values between two consecutive slices were interpolated and therefore discontinuity could be observed from different angles in Movie S1. The volume of the cavity was ~0.34 mm^3^ and the thicknesses of the right ventricle, the septum and left ventricle were ~530 μm, 980 μm, and 1500 μm, respectively (Fig. 4b). The mass could also be estimated with the *a priori* density. Furthermore, valvular and ultra-structures, namely, pectinate muscles in the atrium and trabeculations in the ventricle were visualized (Fig. 4c–e, Movie S2 and S3 in the Supplementary Information). All the pseudo-color in Fig. 4 were based on the gray scale encoded intensity.

### Tracking lineage commitment in a neonatal heart

**Figure 5:**
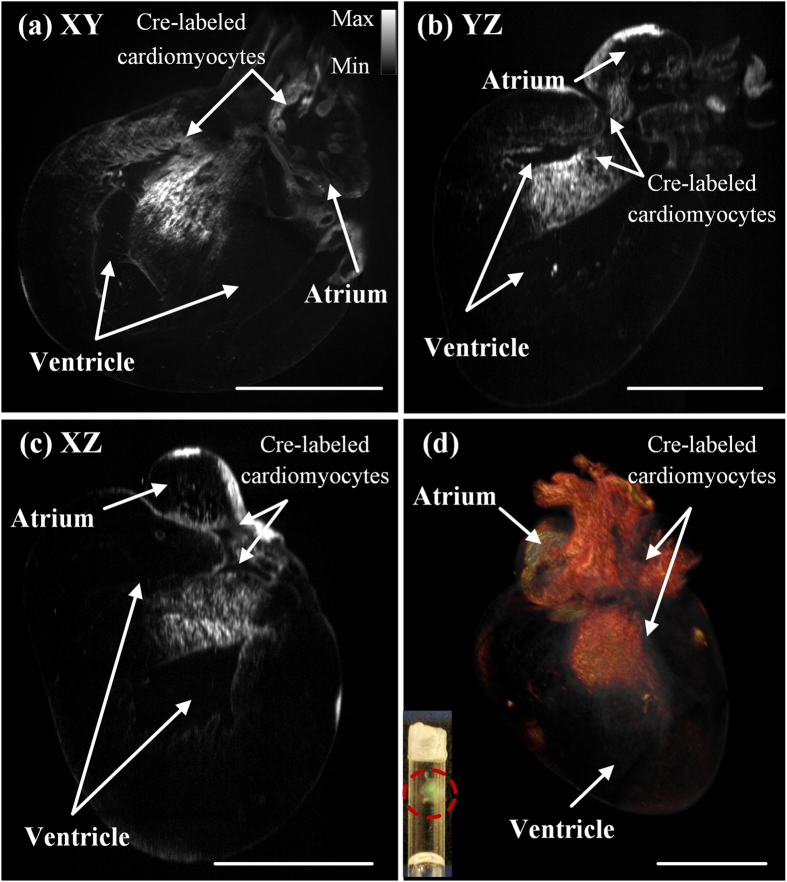
Anatomic structure and cardiac lineage in a P1 (postnatal day 1) mouse heart. (**a–c**) Cross-sectional slices made from the (**a**) *XY*- (**b**) *YZ*- (c) *XZ*-planes reveal the Cre-labeled cardiomyocytes present in both atrium and ventricle. Gray scale allows for encoding the optical intensity. (**d**) 3-D rendering of the reconstructed heart highlights the spatial distribution of Cre-labeled cardiac lineage (see Movie S4 and S5). The inset shows the translucent heart after CLARITY in the tube. Scale bar: 1 mm.

Cardiovascular lineages arise from multipotent progenitors that give rise to diverse cardiac structure and function^32^,^33^,^34^,^35^. Using the lineage specific Cre line as a powerful tool to dissect the lineage commitment of these progenitors enabled, we localized the specific expression of YFP at λ = 532 nm in the atrium and ventricular septum from an atrial specific *Sln*^*Cre/*+^ knockin mouse (P1) with *Rosa26*^*YFP reporter/*+^ gene^36^,^37^ (Fig. 5a,b and c). By stacking all of the slices into a volume at a 5 μm step size, the heart was digitally reconstructed (Fig. 5d). The digital reconstruction allowed for 3-D analyses of the atrial structure and localization of Cre-labeled cardiomyocytes with sufficient spatial resolution and contrast. Thus, large-scale cardiac LSFM demonstrated the capability to track cardiac progenitor differentiation into cardiomyocyte lineages (Movie S4 and S5 in the Supplementary Information). The pseudo-color in Fig. 5 was based on the gray scale encoded intensity. To make the lineage visible (Fig. 5d), the pixel values located outside the user defined intensity threshold were rendered with transparency.

### Localizing the distribution of exogenous potassium channels in an adult heart

**Figure 6:**
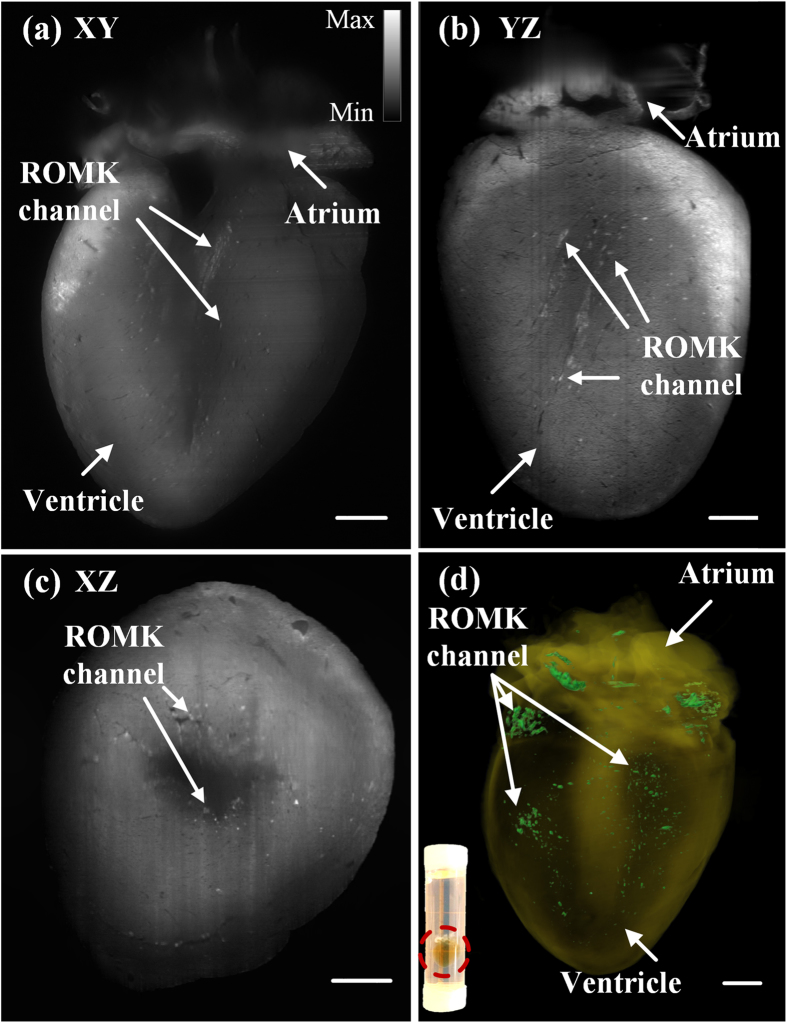
**2-D and 3-D LSFM detection of GFP-tagged ion-channels in a 7.5 month-old adult mouse heart (see** **Movie S6**). (**a–c**) Cross-sectional slices on the (**a**) *XY*- (**b**) *YZ*- (**c**) *XZ*-planes reveal that the spatial distribution pattern of ROMK channels. Arrows indicate the specific fluorescently tagged ROMK channels, and gray scale encodes the optical intensity. (**d**) 3-D rendering of the reconstructed heart contrasts the ROMK channels. The inset shows the translucent heart after CLARITY in the tube. Scale bar: 1 mm.

We have achieved light-sheet fluorescence imaging of an adult murine heart, which is difficult to be fluorescence-friendly cleared and entirely illuminated^26^,^27^. With our large-scale dual-sided illumination light-sheet imaging strategy, we revealed the spatial distribution of renal outer medullary potassium (ROMK) channels after gene modulation/therapy in the intact adult heart as large as 10 by 10 by 10 mm^3^ as illustrated in Fig. 6. Here, an adult mouse heart at 7.5 months of age was imaged in its entirety in 3-D to assess the cardiac-specific expression of an exogenous kidney potassium ion-channel that is otherwise not normally expressed in the heart. The ion-channel signal was excited at λ = 473 nm, and was indicated by arrows after removing stripes in Fig. 6a–c. With the excitation of 473 nm, autofluorescence was still involved in the result. For 3-D reconstruction, we extracted the ion-channel signal by setting intensity threshold in different regions and merged it with pure autofluorescence excited at λ = 532 nm (Fig. 6d, Movie S6 in the Supplementary Information). All the pseudo-color in Fig. 6 was based on the gray scale encoded intensity. To make the distribution pattern visible (Fig. 6d), the pixel values located outside the threshold were rendered with transparency. To validate the results from 3-D LSFM of the adult heart injected with AAV9-cTnT-ROMK-GFP, we also performed GFP-immunostaining to estimate the spatial distribution of ROMK channels. (Figure S2 in the Supplementary Information).

## Discussion

Our large-scale dual-sided illumination cardiac LSFM localizes cardiomyocyte lineage and exogenous ion-channel distribution in intact neonatal and adult murine hearts, respectively. Currently, the barrier in the field of LSFM is the sample size limitation induced by photon scattering and absorption, thus limiting cardiac imaging to very small animal hearts, such as zebrafish and neonatal hearts. To stretch the field’s current sample size limitation to accommodate the larger sizes of the adult murine heart, we have included several features into our large-scale LSFM system: 1) Tuning the relative location of dual-sided illumination beams to simply establish even and effective light-sheet illumination for cardiac imaging. This method allows for imaging neonatal mouse heart by overlapping dual-illumination beams exactly with highest signal-to-background-ratio, and also imaging the whole adult mouse heart by connecting two beams. 2) Improving the CLARITY performance especially for the murine heart. The main differences between our CLARITY method^31^ and the previously published protocol^38^ involve the reduction in clearing reagent use and the simplification of the protocol to eliminate the need for reducing oxygen tension in the sample and thus the need for vacuum pumps or nitrogen gas sources while maintaining sample fidelity and integrity and comparable clearing times. 3) Enhancing the robustness of the system and optimizing the de-noising methodology (Figure S3 in the Supplementary Information) to minimize artifacts. In reference to mSPIM^28^, resonant mirrors are not needed to reduce absorption and scattering artifacts in our system. By using the variational stationary noise remover, our system reduces the complexity of the hardware to enhance the robustness of the entire system. In reference to the light-sheet systems for imaging the large specimens^20^,^21^,^22^, our system has the capacity to image the entire cardiac specimen without the need for stitching image columns. For this reason, our system bypassed the need to move the tissue volume or light-sheet along the propagation of the illumination to meet the beam waist. In addition to visualizing the neural networks^16^, our methodology enhances the robustness of the imaging system to provide the sufficient spatial resolution needed to track cardiac lineage during development (Fig. 5) and potassium channel distribution in the ventricular wall after gene modulation/therapy (Fig. 6). Overall, our methodology simplifies the operation during scanning and reduces the complexity of post-processing for large-scale cardiac imaging. Thus, the integration of our custom-made light-sheet fluorescence microscope, the optimized post-processing methods and the modified CLARITY protocol has advanced the light-sheet imaging strategy for a fundamental direction at the interface of developmental biology and stem cell research.

Despite the longest illumination region to image the entire adult mouse heart, the connection between the two beams is limited to achieve the same signal-to-noise ratio and axial resolution as overlapping dual-sided illumination beams. For the longest illumination region, the illumination along the Z-axis is the sum of one narrow (confocal parameter on one side) and one broad (extension of confocal parameter on the other side) Gaussian beams for the boundary points across the field of view (see right insets in Fig. 1a). As a result, photons from above or below the focal plane of the objective lens would be detected by the sCMOS camera. In comparison to the luminous intensity at the focal plane, the photons that are out of focus contribute primarily to the background signal on the detector, resulting in degradation of the contrast and axial resolution. This influence is present, but minimal for imaging the large-scale adult mouse hearts.

Regarding the spatial resolution, the theoretical optical resolution in the lateral plane (*d*_*lateral*_) is ~1 μm at λ = 532 nm. However, for the experimental results with the 6.3X zoom lens, the measured value (*d*_*lateral*_) is ~2.77 μm as a result of under-sampling from the sCMOS. Despite the zooming ratio of 6.3X, each pixel size (e.g. 6.5 μm) of sCMOS is finally transferred to 1 μm on the sample. However, the digital sampling rate is insufficient to meet the needs of the Nyquist-Shannon sampling theorem in our current cardiac LSFM system. We verified this assumption by measuring the PSF under the same microscope with the wide-field illumination (Figure S4 in the Supplementary Information). For this reason, adopting the over-scanning approach as previously mentioned^25^ would likely reduce information loss from under-sampling.

Compared to single-sided illumination, dual-sided illumination reduces artifacts induced by photon absorption and scattering. However, aligning these two beams confocally is challenging. Moreover, alignment might be degraded to bring about noticeable ghosting or shadowing of the underlying image when the beam penetrates across a large sample. RIMS or glycerol is used to match the refractive index among various interfaces, and to reduce refraction and reflection at the surface between distinct media. Thus, the misalignment or the slight mismatching between two surfaces would lead to a different outcome in the large sample. Assuming an irregular and asymmetric sample with a diameter of 10 mm would engender a coarse misalignment by 0.5°, this misalignment would lead to a vertical displacement by 87 μm (10*tan(0.5°) = 87 μm), which is greater than the axial resolution along the detection path. The misalignment results in integrating two slices onto a single image, thus degrading the axial resolution and causing ghosting or shadowing patterns on the image. Multi-view fusion is an alternative which is able to eliminate these artifacts from misalignment, and enables us to achieve isotropic spatial resolution at the expense of longer acquisition time^39^. Recently, researchers have accelerated the process of multi-view deconvolution, which would require a massive parallel architecture of the graphics processing unit (GPU)^14^,^40^,^41^.

In the application of LSFM for the largest intact heart from a 7.5-month-old adult mouse, we demonstrate the first 3-D visualization of the exogenous potassium channels to supplement conventional visualization by 2-D histological method. Currently, histological evaluation is limited to assessing a few paraffin or frozen sections of 5–7 μm in thickness, followed by extrapolating the limited assessment of the protein distribution from a few 2-D sections to predict the entire heart. Extrapolation is particularly prone to under-sampling if the level of exogenous protein expression (ROMK) is low or asymmetric and the protein distribution is random, clustered, or diffuse. Here, our imaging strategy demonstrates the capacity to track the 3-D distribution of a low level of exogenous protein expression after genetic transduction otherwise non-detectable by histological method. In addition, our imaging system is able to overcome the large-scale adult mouse heart (>10 by 10 by 10 mm^3^ in volume) with deep tissue penetration. While the field of cardiovascular imaging has been able to track the protein distribution in the transgenic mouse heart at the embryo and neonate stages, to our knowledge, we were able to detect even a low level of exogenous protein expression following genetic transduction at the adult stage.

Further optimization of this strategy is warranted to improve spatial resolutions, sampling rate, image contrast, and simultaneous elimination of photo-bleaching to allow for observation of cardiac morphogenesis, regeneration, differentiation and proliferation. Currently, multiple technical developments have been reported. “Non-diffracting” Bessel beam has been employed to generate a much more uniform light-sheet for living organisms with superb axial resolution^10^,^42^, but the field of view is limited to only hundreds of micrometers in such a system. Furthermore, two-photon scanned light-sheet microscopes combining nonlinear excitation^13^,^43^, are ready to improve the imaging depth in highly scattering embryos and to track the dynamic processes *in vivo*. However, these methods have not been directly applied to the large-scale cardiac LSFM as mentioned in imaging of living organisms or embryos. Optimizing the field of view, as well as improving the depth penetration would further enhance the dynamic range for multi-organ imaging. Additionally, optimization of the CLARITY method^31^ may also be critical in minimizing the risk of diminishing or losing fluorescence with prolonged clearing that is necessary for larger hearts.

## Methods and Materials

### Design and construction of the imaging system

Our previous cardiac LSFM focused on small animal models^23^,^24^,^25^, and the new in-house dual-sided illumination light-sheet microscope was built using a continuous-wave laser (LMM-GBV1-PF3-00300-05, Laserglow Technologies, Canada) with triple wavelengths (405 nm: diode laser; 473 nm and 532 nm: diode-pumped solid-state lasers) as the illumination source (Fig. 1). The initial beam width was ~2 mm with a divergence less than 1.5 mrad. All beams emitting from the laser port were aligned to pass through a neutral density filter (NDC-50C-4M, Thorlabs) to a 5x achromatic beam expander (GBE05-A, Thorlabs). An adjustable mechanical slit (VA100C, Thorlabs) was installed to control the incident beam width, and a beam splitter (BS013, Thorlabs) was employed to form dual-illumination, allowing uniform bidirectional sample illumination. Each beam was focused by a plano-convex cylindrical lens (CL1 and CL2: *f* = 50 mm), and was reshaped by a group of achromatic doublets (L1 and L3: *f* = 100 mm; L2 and L4: *f* = 60 mm), resulting in a magnification ratio of 0.6 (L3 and L4). Furthermore, integrating a 2-inch lens (Ob1 and Ob2: *f* = 150 mm) as the objective, with the group of achromatic doublets, allowed for expansion of the beam up to 2.5X times to thoroughly cover the whole sample in width. As a result, the thickness of the light-sheet at the beam waist was ~18 μm with the effective width of 40 mm. Lens parameters are listed in Table 1.

The detection module was installed perpendicular to the illumination plane, and it was composed of a stereo microscope (MVX10, Olympus, Japan) with a 1X magnification objective (NA = 0.25), a scientific CMOS (sCMOS, ORCA-Flash4.0 LT, Hamamatsu, Japan) and a set of filters (Exciter: FF01-390/482/532/640; Emitter: FF01-446/510/581/703; Dichroic: Di01-R405/488/532/635, Semrock, New York, USA). The sample immersed in the refractive index (RI) matching solution (RI: 1.46–1.48^44^) with 1% agarose solution was mounted in a Borosilicate glass tubing (RI = 1.47^45^, Pyrex 7740, Corning, New York, USA) to reduce refraction and reflection among various interfaces (Fig. 1). This glass sample holder was placed in a 3-D-printed opening chamber, made of acrylonitrile butadiene styrene (ABS) (uPrint, Stratasys, USA) and filled with 99.5% glycerol (RI = 1.47^45^). A piece of cover glass (RI = 1.47^45^, Premium, Fisher Scientific, USA) was embedded on each side of the chamber, perpendicular to the illumination beam to minimize refractive index mismatching. During scanning, the sample holder was oriented and moved by a motorized 3-D translational stage. Both illumination and detection modules were controlled by a computer with dedicated SSD RAID0 storage for fast data streaming.

### Data acquisition and image post-processing

The lateral resolution of this cardiac LSFM is mainly governed by the NA of objective and is denoted by *d*_*lateral*_ = 0.61*λ*/*NA*, where *λ* indicates the wavelength of excitation light. The axial resolution is determined by the waist of Gaussian beam and detection NA. In general, a cylindrical lens generates a hyperbolic pattern instead of a plane of light. The waist *ω*_*0*_ and Rayleigh range *Z* (or confocal parameter, 2*Z*) are used to define the light-sheet^19^: *ω*_0_ = *λf*/*πω*, *Z* = *λf*^2^/*πω*^2^, where *f* is the focal length of excitation objective, and *ω* denotes the half of the width of illumination beam before focusing. Within this Rayleigh range *Z*, the object is approximately sectioned by the light-sheet with constant thickness 2*ω*_*0*_. Based on these equations, both waist *ω*_*0*_ and Rayleigh range *Z* drop as the *ω* increases. By controlling the slit size and overlap region of the two beams, the parameter *ω*_*0*_ varies from nearly 9 μm to 50 μm, while *Z* ranges from hundreds of micrometer to tens of millimeters.

During data acquisition, the detection objective imaged through the liquid-air interface. Each image was acquired within 50 ms exposure time. The stepping size of mechanical scanning was 1~5 μm, smaller than one half of the light sheet thickness in accordance with Nyquist-Shannon sampling theorem. The translational stage moved steadily to avoid acceleration or deceleration. The optical magnification varied from 0.63X to 6.3X, leading to a lateral pixel size of ~10 μm to 1 μm (sCMOS pixel size: 6.5 μm). Thus, the digital resolving power of the cardiac LSFM in cross-section varied from 1 μm to 10 μm. All of the raw data were processed to remove stationary noise^29^,^30^. The 3-D rendering and image segmentation were processed by Amira 6.1 (FEI Software).

### Fluorescent beads preparation for LSFM calibration

For testing the illumination region and PSF, fluorescent polystyrene beads (Spherotech Inc) with 0.53 μm diameter were used. The concentration of the bead solution was diluted to 1:150000, and the beads were embedded in RIMS with 1% low-melt pointing agarose in a Borosilicate glass tubing (Pyrex 7740, Corning, New York, USA). All of these components were immersed inside the chamber filled with glycerol or RIMS to provide optical clarity for fluorescence detection.

### Post-natal mouse heart imaging: wild type vs. *Sln* ^*Cre/*+^; *R26* ^*YFP reporter/*+^ double heterozygous strain

Animal protocols, experiments, and housing were approved by the University of California Los Angeles institutional review committees (IACUC) in compliance with the Guide for the Care and Use of Laboratory Animals published by the US National Institute of Health. New born mouse hearts (wild type) were dissected and imaged at postnatal day 1 (P1) and 7 (P7). Mouse hearts were also prepared from crossing the *Sln-Cre* knockin strain (*Sln*^*Cre/*+^) with Rosa26-YFP reporter strain (Jax) to generate *Sln*^*Cre/*+^; *R26*^*YFP reporter/*+^ double heterozygous mice.

### Adult mouse heart imaging: transmembrane potassium channels

An adult mouse heart of 7.5 months of age was imaged at 5.5 months upon tail vein injection of 8.7 × 10^12^ viral genomes (vg) of adeno-associated virus vector 9 (AAV9; Vector Biolabs). This vector system employed a cardiac-specific troponin T promoter (cTnT) to drive cardiomyocyte gene expression of a customized construct (UCLA Cardiovascular Research Laboratory) in which the renal outer medullary potassium (ROMK) channel was fused at its C-terminus to one green fluorescent protein (GFP) molecule (AAV9-ROMK-GFP).

### Chemical clearing

The hearts were rinsed in phosphate-buffered saline (PBS) three times for 10 minutes and fixed in 4% paraformaldehyde (Electron Microscopy Sciences) at 4 °C overnight. Next, the samples were placed in a 4% acrylamide solution along with 0.5% w/v of the photoinitiator 2,2′-Azobis[2-(2-imidazolin-2-yl)propane]dihydrochloride (VA-044, Wako Chemicals USA), followed by overnight incubation at 4 °C and incubation at 37 °C for 2–3 hours to initiate polymerization of the acrylamide. Once polymerized, the tissues were rinsed with PBS and placed into a clearing solution comprised of 8% w/v sodium dodecyl sulfate (SDS, Sigma Aldrich, USA) and 1.25% w/v boric acid (Fischer, USA) (pH = 8.5). The samples were incubated at 37 °C until they were clear, followed by one day in 1x PBS to remove residual SDS. The hearts were incubated in RIMS until transparent. Additionally, RIMS was made of forty grams of Sigma D2158 (Histodenz) in 30 ml of 0.02 M PB with 0.1% tween-20 and 0.01% sodium azide, pH to 7.5 with NaOH.

### Immunohistochemistry to validate spatial distribution of ROMK channels

Immunohistochemical staining of GFP was performed on 5-μm formalin-fixed paraffin sections prepared from AAV9-cTnT-ROMK-GFP transfected adult hearts using the Envision + System Horseradish Peroxidase (HRP) anti-rabbit secondary antibody K4002 (Dako) conjugated to sc-8334, a primary antibody against GFP (Santa Cruz Biotechnology). For each heart level examined, negative control sections were obtained by omitting the primary antibody sc-8334 and incubating with only the secondary antibody K4002. ROMK channel expression level and distribution pattern were assessed by identifying the brown ROMK-GFP positive myocytes using QIClick Digital CCD Camera (QImaging, Surrey, BC, Canada) installed on a Leica DM5500 B microscope (Leica Microsystems CMS GmbH, Wetzlar, Germany) that is equipped with OASIS-Glide motorized stage and Turboscan slide-scanning for rapid, accurate seamless mosaic stitching (Surveyor software, Objective Imaging Ltd, U.K.).

## Additional Information

**How to cite this article:** Ding, Y. *et al*. Light-sheet fluorescence imaging to localize cardiac lineage and protein distribution. *Sci. Rep.* **7**, 42209; doi: 10.1038/srep42209 (2017).

**Publisher's note:** Springer Nature remains neutral with regard to jurisdictional claims in published maps and institutional affiliations.

## Supplementary Material

Supplementary Movie S1

Supplementary Movie S2

Supplementary Movie S3

Supplementary Movie S4

Supplementary Movie S5

Supplementary Movie S6

Supplementary Information

## Figures and Tables

**Table 1:** Parameters of Optical Components.

Item	*f* (mm)	Component	Manufacturer
CL1, CL2	50	LJ1695RM-A	Thorlabs
L1, L3	100	AC254-100-A	Thorlabs
L2, L4	60	AC254-060-A	Thorlabs
Ob1, Ob2	150	AC508-150-A	Thorlabs
