# Supplementary material for: Light-sheet fluorescence imaging to localize cardiac lineage and protein distribution
Source: Sci Rep. 2017 Feb 6;7:42209. doi: 10.1038/srep42209 (PMC5292685; doi:10.1038/srep42209)
Supplement: Supplementary Information [file srep42209-s7.pdf]

Supplementary Information

Light-sheet fluorescence imaging to localize cardiac lineage and protein distribution

Yichen Ding[1,2,§], Juhyun Lee[2,§], Jianguo Ma[1,§], Kevin Sung[2], Tomohiro Yokota[3], Neha Singh[1], Mojdeh Dooraghi[1], Parinaz Abiri[1,2], Yibin Wang[3], Rajan P. Kulkarni[2,6,7], Atsushi Nakano[4,5], Thao P. Nguyen[1], Peng Fei[8] and Tzung K. Hsiai[1,2,7,*]

[1] Division of Cardiology, Department of Medicine, UCLA, Los Angeles, CA 90095, USA
[2] Department of Bioengineering, School of Engineering & Applied Sciences, UCLA, Los Angeles, CA 90095, USA
[3] Departments of Anesthesiology, Physiology and Medicine, Cardiovascular Research Laboratories, UCLA, Los Angeles, CA 90095, USA
[4] Department of Molecular, Cellular and Developmental Biology, UCLA, Los Angeles, CA 90095, USA
[5] Eli and Edythe Broad Center of Regenerative Medicine and Stem Cell Research, UCLA, Los Angeles, CA 90095, USA
[6] Division of Dermatology, Department of Medicine, UCLA, Los Angeles, CA 90095, USA
[7] California NanoSystems Institute, UCLA, Los Angeles, CA 90095, USA
[8] School of Optical and Electronic Information, Huazhong University of Science and Technology, Wuhan, China

[§]These authors contributed equally to this work.

*Corresponding Author
Email: THsiai@mednet.ucla.edu

This PDF file includes:

Figure S1

Figure S2

Figure S3

Figure S4

In **Figure S1**, to verify that all the values in **Figure 3c** of the main article were the thickness of light-sheet at different regions, we further measured the axial PSF of the detection objective (Olympus, MVPLAPO 1X) under the wide-field illumination, and compared with the results of light-sheet illumination with variable slit size. We demonstrate that the values in aforementioned **Figure 3c** refer to the thickness of light-sheet instead of axial PSF of the detection objective.

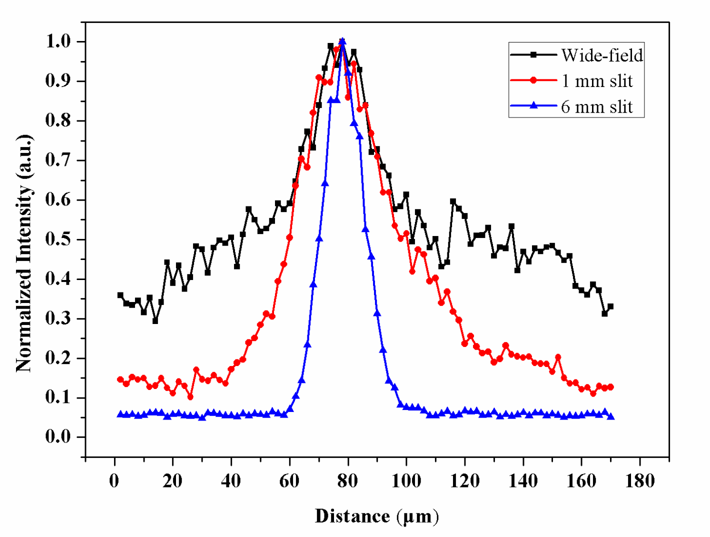

Figure S1. Comparison of axial resolutions under the condition of wide-field illumination and light-sheet illumination with variable slit sizes.

To validate that the fluorescence pattern in **Figure 6** of the main article is specific to that of the GFP-labeled ROMK expression, we performed GFP-immunostaining to estimate the spatial distribution of ROMK channels. GFP immunostaining was performed on 5-μm thick paraffin sections from the heart of a 16-month-old mouse at 4 months upon tail vein injection with AAV9-ROMK-GFP. In the column 1 of **Figure S2**, the exogenous fusion ROMK-GFP construct is expressed as a transmembrane ion channel tetramer (arrows) composed of four identical subunits with each subunit linked at its cytoplasmic C-terminus to one GFP molecule, identified by the brown immunostaining, in these right atrial sections. In the column 2, negative controls (obtained by omitting the primary antibody sc-8334 and incubating with only the secondary antibody K4002) failed to reveal any GFP immunostaining, similar to the lack of GFP immunolabeling in heart sections of mice not receiving viral injection.

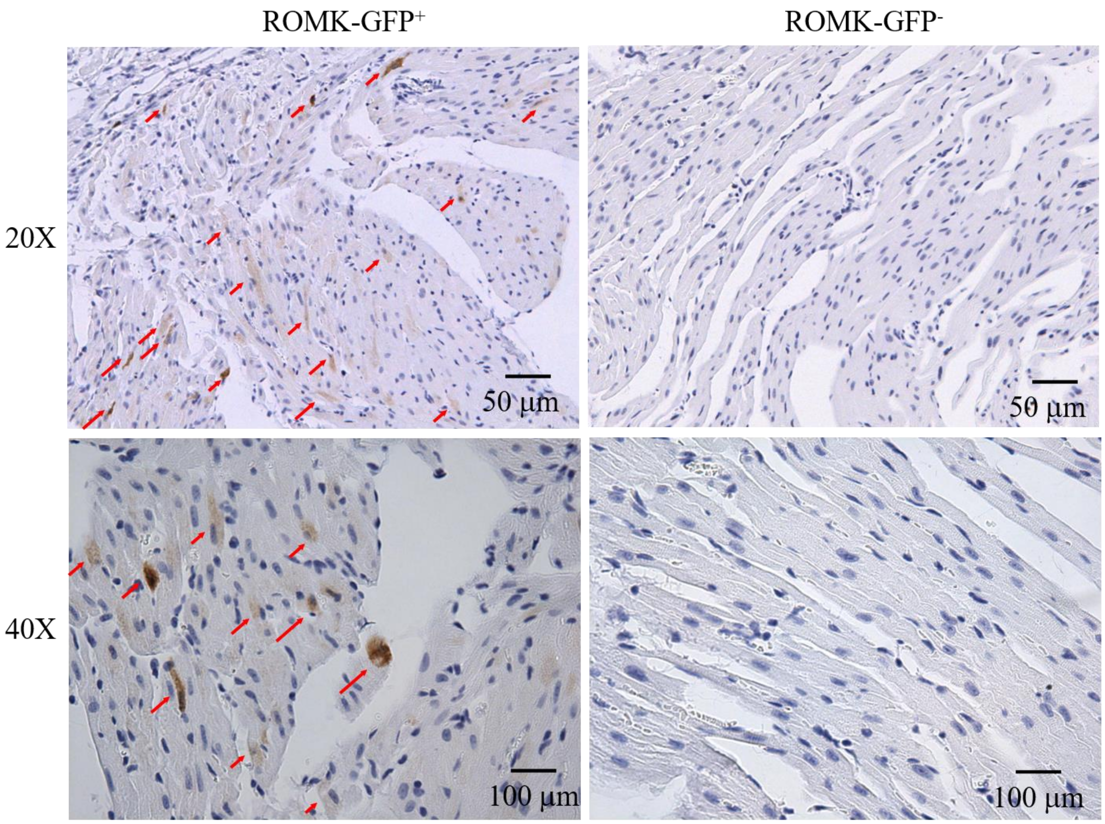

Figure S2. Exogenous ROMK expression in the adult heart identified by immunohistochemistry to confirm the authenticity of GFP fluorescence by LSFM. Scale bar: 50 μm (top) and 100 μm (bottom).

For the post-processing approach, the variational stationary noise remover (VSNR) was reported [1,2]. Here, we further optimized light-sheet imaging for the mouse heart. In **Figure S3**, we applied different settings (sigma_x, sigma_y, lambda, angle, phase and noise level) to demonstrate the distinct VSNR results. The **Figure S3a** presents the raw data, while **Figure S3b** provides the optimized result. The stripes (red arrow) in the zoom-in regions in **Figure S3a** were filtered in **Figure S3b**. However, using the previously reported setting [1,2] in **Figure S3c** brings about artificial shadows (red arrow). We also reveal the invalid case which failed to remove artificial stripes (**Figure S3d**), and the aggressive cases which degraded the original signal (**Figure S3e** and **S3f**) in order to highlight the essence of proper filter parameters.

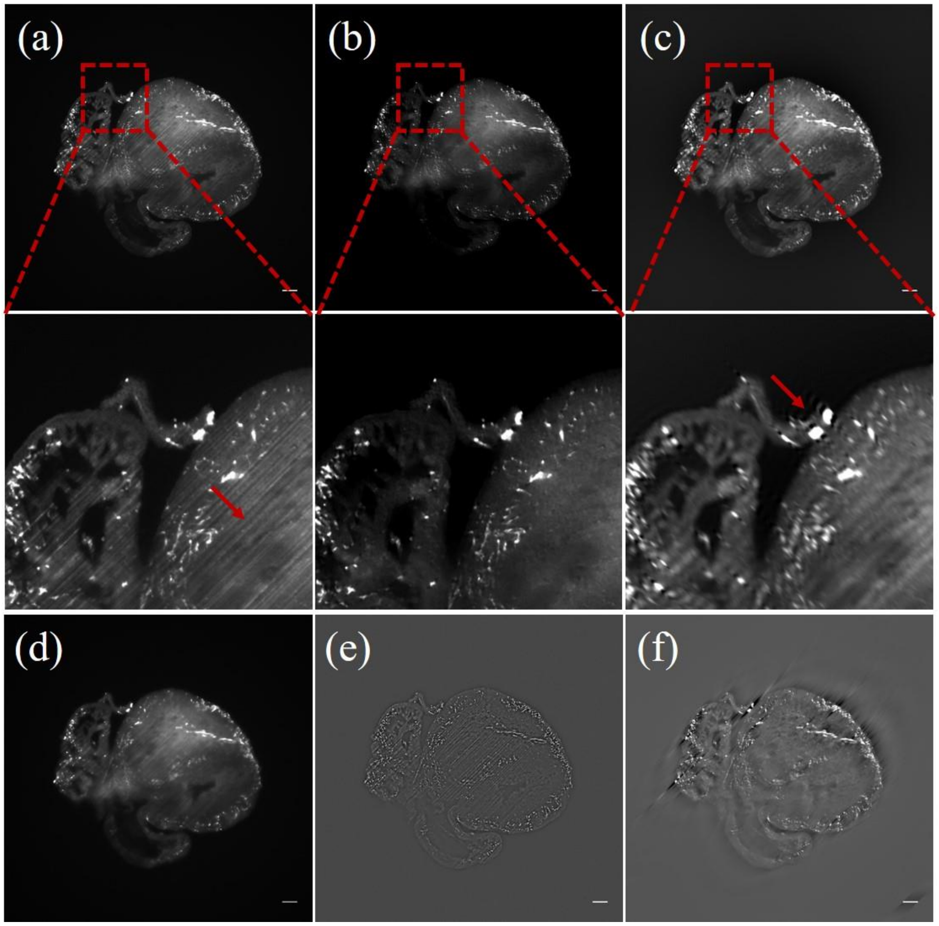

Figure S3. Comparison of different VSNR settings. Demo image shows a single slice of a mouse heart. (a) Raw data. (b) Optimized result. (c) Default result. (d) Invalid result. (e-f) Aggressive results. Scale bar: 200 µm.

In the current system, the theoretical optical resolution was ~1 µm given by a 0.25 NA objective. Considering the highest camera sampling rate was 1 µm in the lateral plane, the recorded image would provide 2 µm lateral resolution at most based on the Nyquist-Shannon sampling theorem. To verify the resolution loss was not induced by spherical aberration, we placed the 0.53 µm fluorescent beads on the cover glass and captured two images under ordinary microscopes, one was wide-filed image with the objective (Olympus, MVPLAPO 1X, NA=0.25) used in the light-sheet fluorescent microscope (**Figure S4a**), and the other one was under the commercial fluorescence microscope (Zeiss Observer. Z1) with the oil immersion objective (Zeiss, Plan Apochromat, 63X, NA=1.4) (**Figure S4b**). First, the high magnification image showed the bead nearly 0.5 µm (**Figure S4b**), and therefore, it was adequately small to be used for measuring PSF in our light-sheet imaging system. We measured the FWHM of the wide-field image without light-sheet illumination and liquid solution, and we showed the comparable lateral resolution (FWHM=2.92 µm in **Figure S4c**) as described in the main article.

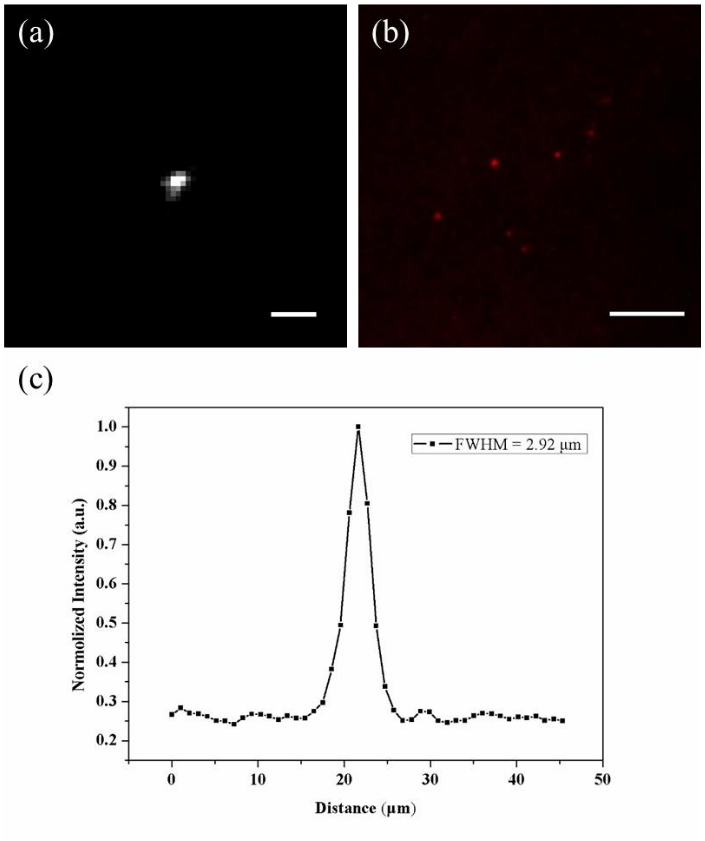

Figure S4. (a) Wide-field imaging result of a single bead. (b) Commercial fluorescence imaging of a single bead. (c) The lateral resolution of the objective (Olympus, MVPLAPO 1X) without spherical aberration and liquid solution. Scale bar: (a) 10µm; (b) 5µm.

Other Online Supporting Information for this manuscript includes the following:

Movie S1. 3-D rendering of the reconstructed P7 heart
Movie S2. Pectinate muscle in the right atrium
Movie S3. Trabecular muscle in the ventricle
Movie S4. 3-D rendering of the reconstructed heart
Movie S5. 3-D rendering of the reconstructed heart with slices
Movie S6. 3-D rendering of GFP-tagged ion-channels in a 7.5 month-old adult mouse heart

References

1. Fehrenbach, J., Weiss, P. & Lorenzo, C. Variational algorithms to remove stationary noise: applications to microscopy imaging. *IEEE T. Image Proc.* **21**, 4420-4430 (2012).
2. Fehrenbach, J. & Weiss, P. Processing stationary noise: Model and parameter selection in variational methods. *SIAM J. Imag. Sci.* **7**, 613-640 (2014).
